# Oxidative Stress and Air Pollution Exposure

**DOI:** 10.1155/2011/487074

**Published:** 2011-08-13

**Authors:** Maura Lodovici, Elisabetta Bigagli

**Affiliations:** Department of Pharmacology and Toxicology, University of Florence, Viale Pieraccini 6, 50139 Florence, Italy

## Abstract

Air pollution is associated with increased cardiovascular and pulmonary morbidity and mortality. The mechanisms of air pollution-induced health effects involve oxidative stress and inflammation. As a matter of fact, particulate matter (PM), especially fine (PM_2.5_, PM < 2.5 **μ**m) and ultrafine (PM_0.1_, PM < 0.1 **μ**m) particles, ozone, nitrogen oxides, and transition metals, are potent oxidants or able to generate reactive oxygen species (ROS). Oxidative stress can trigger redox-sensitive pathways that lead to different biological processes such as inflammation and cell death. However, it does appear that the susceptibility of target organ to oxidative injury also depends upon its ability to upregulate protective scavenging systems. As vehicular traffic is known to importantly contribute to PM exposure, its intensity and quality must be strongly relevant determinants of the qualitative characteristics of PM spread in the atmosphere. Change in the composition of this PM is likely to modify its health impact.

## 1. Introduction

Numerous epidemiological studies have shown an increased morbidity and mortality due to environmental air pollution [1, 2]. Environmental air does contain a complex mixture of toxics, including particulate matter (PM), irritant gases, and benzene. The chemical composition of particles does vary greatly and depends on numerous geographical, meteorological, and source-specific variables. Generally, environmental particles include inorganic components (sulfates, nitrates, ammonium, chloride, and trace metals), elemental and organic carbon, biological components (bacteria, spores, and pollens), and adsorbed volatile and semivolatile organic compounds [3]. In addition, environmental particles, when mixed with atmospheric gases (ozone, sulfur nitric oxides, and carbon monoxide) can generate environmental aerosols. Particles are usually defined as PM_10_ and PM_2.5_ with diameter less than 10 and 2.5 *μ*m, respectively. Any fraction may have different effects; that is, PM with aerodynamic diameter less than 10 to 2.5 *μ*m does generate a bigger amount of hydroxyl radical due to the heavy metals adsorbed on the pores and surfaces of the particles, whereas particles of larger size (PM_10_) deposit mainly in the upper airways and can be cleared by the mucociliary system [4, 5]. Recently, however, interest has also focused on the ultrafine particles (UFPs) with a diameter less than 100 nm; UFPs are considered important with respect to health effects because of their very high alveolar deposition fraction, large surface area, chemical composition, and ability to enter into the circulation and induce inflammation. Vehicle emissions, in particular related to diesel engines, diesel exhaust particles (DEPs), are a major source of environmental UFPs, which in the presence of poor ventilation may penetrate indoor, where additional sources including environmental tobacco smoke, cooking, burning of candles, and chemical reactions are present [6–10]. Long-term exposure to high levels of such particles can increase risk of cancer, respiratory diseases, and arteriosclerosis, whereas short-term exposure peaks can cause exacerbation of bronchitis, asthma, and other respiratory diseases as well as changes in heart-rate variability [2, 11–13]. The general consensus does indicate that the mechanism of air pollution-induced health effects involves an inflammation-related cascade and oxidation stress both in lung, vascular, and heart tissue [14–19]. Inflammation is initially a protective mechanism which removes the injurious stimuli and produces reactive oxygen species (ROS) able to induce cell killing. In the early phase of inflammation, oxidant stress does not directly cause cell damage and can induce the transcription of stress defense genes including antioxidant genes. This preconditioning effect of ROS enhances the resistance against future inflammatory oxidant stress and promotes the initiation of tissue repair processes. The additional release of cell contents amplifies the inflammatory process and consequently can induce tissue injury [20]. Oxidation damage has been implicated in many degenerative and nondegenerative diseases, including cardiovascular and pulmonary diseases, diabetes, and Alzheimer disease. Oxidation stress derived from an unbalance between ROS formation and individual antioxidant activity potentially does lead to damage of lipids, proteins, and macromolecules such as DNA and RNA [21]. This paper will focus on the mechanisms of oxidative stress induction and cellular damage by air pollution exposure on pulmonary and cardiovascular systems.

## 2. Possible Mechanisms of Oxidative Stress Induced by Air Pollution Exposure

In the last decades, great attention has been paid to air pollution exposure due to vehicular traffic and other combustion processes. PM and gas pollutants are considered to be the most important factors in urban areas, and several mechanisms have been hypothesized to explain the adverse health effects in humans, especially in the cardiopulmonary system [22]. Although each air pollutant can exert its own specific toxicity in the respiratory and cardiovascular systems, ozone, oxides of nitrogen, and suspended particulates all share a common property of being potent oxidants, either through direct effects on lipids and proteins or indirectly through the activation of intracellular oxidant pathways [23–25].

ROS can be generated from the surface of particles where polycyclic aromatic hydrocarbons (PAH) and nitro-PAH are adsorbed, other than transition metals (iron, copper, chromium, and vanadium) that catalyzing Fenton's reaction (Fe^2+^ + H_2_O_2_ + H^+^ → Fe^3+^+ OH^•^ + H_2_O) generate the highly reactive hydroxyl radical able to induce oxidative DNA damage [26, 27]. Several studies have shown that iron and other transition metals leaching from particles or by their presence on particle surfaces play a role in the generation of ROS in biological systems [28]. Particles bound benzo(a)pyrene has been shown to be bioavailable and can induce oxidative DNA damage in systemic target organs, including lung and kidney [29, 30]. Moreover, it should be noted that ozone and nitrogen dioxide are usually present together with particles in environmental air. They are also oxidants with potential effects in terms of oxidative DNA damage. Similarly, volatile compounds, such as benzene, in urban air pollution can induce DNA oxidation [31, 32]. In addition, photochemical oxidants (ozone and peroxyacetyl nitrate), secondary pollutants formed by the action of sunlight on an atmosphere that does contain reactive hydrocarbons and NOx, contribute to increase oxidation stress [33]. Then, in the presence of high ROS formation, mitochondrial damage with induction of NADPH- oxidase isoform 4 (NOX4) does occur, together with an activation of inflammatory cells (neutrophils, eosinophils, and monocytes) and increased numbers of macrophages capable of ROS and reactive nitrogen species generation [34–36]. Initially, when oxidative stress is relatively low, various transcription factors, such as the nuclear factor erythroid-2 (Nrf2), induce a series of antioxidant and detoxification enzymes (e.g., catalase, superoxide dismutase, and glutathione S-transferase) that counteract ROS formation protecting from adverse biological outcomes [37, 38]. In the second phase, if the protective antioxidant response fails or is inadequate to deal with increasing ROS production, the result is a proinflammatory situation with various cytotoxic effects [39]. These effects are mediated by the redox-sensitive mitogen-activated protein kinase (MAPK) and NF-*κ*B cascades that are responsible for the expression of cytokines, chemokines, and adhesion molecules, which are involved in inflammatory processes [39].

## 3. Atmospheric Gases

Gaseous pollutants contribute to a great extent in composition variations of the atmosphere and are mainly due to combustion of fossil fuels and to emission of motor vehicles [40].

Ozone is a strong oxidizing agent formed in the troposphere through a complex series of reactions involving the action of sunlight in nitrogen dioxide and hydrocarbons. Ozone initiate intracellular oxidative stress through ozonide and hydroperoxide formation. This mechanism of oxidative damage involves the activation of Nrf2, heat shock protein 70, NF-*κ*B, increased expression of a range of proinflammatory cytokines (TNF*α* and interleukin 1*β*), chemokines (e.g., interleukin 8), and adhesion genes; ozone is also an activator of protein-1 fos and c-jun onco genes [41, 42]. The major source of anthropogenic emissions of nitrogen oxides into the atmosphere is the combustion of fossil fuels deriving from stationary sources (heating, power generation) and motor vehicles. In environmental conditions, nitric oxide is rapidly transformed into nitrogen dioxide by atmospheric oxidants such as ozone [43].

Various antioxidants, like ascorbic acid, uric acid, and thiols, act as powerful scavengers of O_3_ and NO_2_^•^ radical in body fluids, likely protecting lung lining fluids against inhaled oxidizing air pollutants [44]. When such defense mechanisms are overwhelmed, O_3_ may injure the underlying cells by inducing lipid peroxidation and activating inflammatory gene expression [45]. *In vitro* and *in vivo* studies, both in animals and human beings, confirm the capacity of nitrogen dioxide to activate oxidant pathways although less potently than ozone [46]. Volatile organic compounds are a class of compounds which includes chemical species of organic nature such as benzene, but the majority of gaseous pollutants are inhaled and, therefore. mainly affect the respiratory and cardiovascular systems. Among gaseous pollutants, carbon monoxide (CO) has been described as one of the main pollutants responsible for the development of cardiovascular diseases [47], while benzene can also induce haematological problems and cancer [48]. 

Benzene is a commonly used industrial chemical and a constituent of gasoline [31]. Inhalation is the most important route of absorption during occupation-related exposure. Benzene toxicity is attributed to its metabolism, which does lead to the formation of reactive metabolites such as hydroquinone and its oxidized form benzoquinone which are highly reactive molecules and, by means of redox cycling, produce ROS [49]. Furthermore, the addition of antioxidant enzymes has been shown to block oxidative damage induced by the above-mentioned metabolites confirming the role of ROS production and oxidative stress in hydroquinone and benzoquinone cytotoxicity [50]. Uzma et al. [31] demonstrated that occupation-related exposure to benzene causes oxidative stress, immune suppression, and inducing the expression of tumour-suppressing gene p53 in gasoline filling workers. These authors hypothesized that the increase in the p53 expression may block the cell cycle at G1 phase and go on to repair DNA damage, which is the initial step in tumour suppression.

## 4. Oxidative Stress from Organic Fraction

Ambient PM, does consist of complex and various mixtures of particles suspended in the breathing air [50]. Major sources of PM are factories, power plants, refuse incinerators, motor vehicles, building activity, fires, and natural windblown dust. The size of the particles vary, and there is strong evidence supporting that ultrafine and fine particles are more hazardous than larger ones in terms of mortality and cardiovascular and respiratory effects [51].

Results from various surveys have demonstrated that oxidative potential of fine and ultrafine particles is the result of significant amounts of organic carbon compounds, such as quinones and PAHs. In the organic fraction originating in the air from incomplete combustion processes, the major reactive and toxic compounds are substituted (e.g., methyl naphthalene) and unsubstituted PAH, nitro-PAH (1-nitropyrene and 3-nitro-fluoranthene), dinitro-PAH (dinitro pyrene) and peroxyacetyl nitrate [52, 53]. Moreover, reactive intermediates in the oxidation of mixtures of volatile organic compounds (VOCs), oxides of nitrogen (NOx), hydroxy radical, and ozone are shown to play a central role in the formation and fate of airborne toxic chemicals, PAH, and fine particles [52]. The main pathways of metabolic activation of PAHs are generation of diol epoxides catalyzed by cytochrome P450 (CYP450), leading to DNA adduct formation, formation of radical cations catalyzed by CYP450 peroxidases, and formation of redox-active quinones [54]. Valavanidis et al. [55] demonstrated that redox-active transition metals, redox cycling quinoids and PAH act synergically to produce ROS. J. Y. C. Ma and J. K. H. Ma [56] reported that organic fraction of DEP, mainly constituent of PAH and quinones, does undergo to metabolic activation in the lung and liver of exposed animals, is able to induce CYP4501A1 isoform expression that generates ROS and reactive PAH-quinones. In addition, PM initiates inflammatory damage upregulating proinflammatory cytokines and chemokines; *in vitro* observations have shown that PM exposure may cause expression of nuclear factor NF-*κ*B-related genes and oxidant-dependent NF-*κ*B activation [57, 58]. To defend against oxidative damage, cells increase the production of antioxidant enzymes through the activation of the Nrf2, [37] and PM appears to inhibit protective enzymes involved in oxidative stress responses leading to the activation of additional intracellular signaling cascades that regulate the expression of cytokine and chemokine genes [59]. Many recent observations have shown that DEPs, because of their fine and ultrafine composition, play an important role on oxidative cellular damage through ROS generation causing lipid peroxidation and oxidative DNA damage. Some DEPs consist of a carbon core or organic droplets with adsorbed organic compounds, such as PAH, quinines, and redox-active metals. The capacity of DEPs to induce oxidative stress is largely related to these adsorbed components [60, 61].

## 5. Oxidative Stress Induced by Transition Metals

Transition metals such as iron, lead, mercury, cadmium, silver, nickel, vanadium, chromium, manganese, and copper are detectable in PM_2.5_ and UFPs adsorbed on their surface and are capable of ROS formation by Fenton's reaction [35]. As critical constituents of PM, transition metals were postulated to be involved in a number of pathological processes of the respiratory system through free radical-mediated damage [62]. They are natural components of the earth's crust and enter into the environment through a wide variety of sources, including combustion, waste water discharges, and manufacturing facilities. Iron is a well-known soot suppressant that might be emitted into the atmosphere in the form of ultrafine particles [63]. Zinc is a major metal element detected in traffic derived PM_2.5_, deriving from waste oil samples [64]. Copper is a component of car brake pads, however, ceramic brake pads contain 10%–20% copper by mass, while the metallic brake pads contained about 70% iron with very little copper. This metal in PM has also been linked to road traffic sources associated to PM_2.5_ [64]. Soluble metals in inhaled particles, such as Fe, Ni, V, Co, Cu, and Cr, were associated with increased ROS production, followed by cellular oxidative stress in airway epithelial cells [65].

## 6. Air Pollution Induced-Oxidative Damage in Target Organs: Cardiovascular and Pulmonary Systems

### 6.1. Cardiovascular System

Diesel and gasoline vehicle emissions in the urban areas have dominant contributions to environmental particles, especially those located in the ultrafine range. Because of their small size and large surface area, UFPs have demonstrated unique biochemical characteristics, such as enhanced ability to adsorb or absorb organic molecules and to penetrate into cellular targets in the human pulmonary and cardiovascular systems [66, 67]. UFPs may be directly transported to the cardiac vasculature, where they can induce arrhythmias, reduce myocyte contractility, and decrease coronary blood flow [10, 68]. Studies by Brook et al. [69] demonstrated that fine particulate air pollution and ozone cause acute arterial vasoconstriction in healthy humans, while Urch et al. [70] reported that fine particles exposure pollution raise blood pressure and impair vascular function. In addition, UFP exposure depresses myocardial contractile response and coronary flow in both spontaneously hypertensive and wild-type rats [71], the same observation was found by Simkhovich et al. [72] in young and old rat hearts. Long-term exposure to low concentrations of PM_2.5_ has been shown to alter vasomotor tone, lead to vascular inflammation, and potentiate atherosclerosis induced by highly fat-containing chow in susceptible mice [73]. In addition, Suwa et al. [74] reported that exposure to PM_10_ cause progression of atherosclerotic lesions towards a more advanced phenotype hyperlipidemic rabbits. Moreover, atherosclerotic lesions of thoracic aorta were reported to be significantly increased with pronounced macrophage infiltration and lipid deposition in Apolipoprotein E (−/−) ApoE (−/−) mice exposed to PM_2.5_ through NADPH oxidase dependent pathways [75]. ApoE (−/−) mice exposed to ozone showed increased oxidative stress and mitochondrial DNA damage, decreased vascular endothelial nitric oxide synthase, and significantly increased atherogenesis compared to filtered air exposed controls [76]. Recently, Cherng et al. [77] reported that DEP exposure enhances vasoconstriction and diminishes acetylcholine-induced dilatation in coronary arteries of animals in a nitric oxide synthase-dependent manner. Baccarelli et al. [78] showed that air pollution is associated with changes in the global coagulation function, after short-term exposure to air pollution in normal subjects resident in Lombardia Region, Italy. Road traffic-related pollutants may increase a heart-rate-corrected QT interval among people with diabetes, obesity and nonsmoking elderly individuals and the number of genetic variants related to oxidative stress does increase this effect [79]. On the contrary, Mordukhovich et al. [80], despite the positive associations between blood pressure and black carbon, found no effects on gene variants related to antioxidative defense. Increases in black carbon and PM_2.5_ were associated with increases in blood pressure, heart-rate, endothelin-1, vascular endothelial growth factor, and oxidative stress markers and with a decrease in brachial artery diameter in nonsmoking seniors [81]. More recently, Kooter et al. [82] showed that diesel engine exhaust exposure induces a pulmonary antioxidant response, with an increased activity of the anti-oxidant enzymes glutathione peroxidase, superoxide dismutase, heme oxygenase-1 protein, heme oxygenase activity, and uric acid which precedes the inflammatory response (an increase in IL-6 and TNF-**α**) in rats. In addition, since the authors found that increased plasma thrombogenicity and antioxidant defense gene expression in aorta tissue shortly after the exposure does occur, they hypothesized a direct translocation of diesel engine exhaust components to the vasculature even if the mediation by other pathways cannot be excluded [82]. 

### 6.2. Pulmonary

A strong correlation has been found between PM concentration of redox-active compounds and damage in macrophages and bronchial epithelial cells [83–85]. Moreover, in human airway epithelial cells, organic compounds adsorbed on particle surfaces does promote inflammation through CYP1A1-mediated ROS generation and release of cytokines after activation of transduction pathways involving MAPK and the transcription factor NF-kappaB [86]. Recently, Andersson et al. [26] reported that 1-nitropyrene, one of the most abundant nitro-PAHs in diesel exhausts, induces DNA damage by ROS formation in human endothelial cells, and this effect was mainly mediated by metabolites mainly generating by reduction of nitro group, as it has been previously reported by Topinka et al. [87] in rat hepatocytes. Increased production of ROS after PM exposure is suggested by the finding that many of the proinflammatory genes (TNF-*α* and IL-8, among others) induced upon exposure to PM are regulated by redox sensitive transcription factors such as NF-*κ*B, activator protein 1 (AP-1) and CAATT/enhancer binding protein (C/EBP). Activation of these transcription factors and increased transcription of downstream genes has been reported in human alveolar and bronchial epithelial cells in response to PM exposure [88–92]. Several studies have demonstrated that air pollution particles induce inflammatory mediator release and oxidative stress in lung epithelial cells and alveolar macrophages. When reaching the bone marrow [93], cytokines and chemokines released from the lung stimulate migration of neutrophils and their precursors into the circulation. In the short-term, there is acute tissue damage with activation of the epidermal-growth-factor receptor pathway, and evidence for organ-repair responses [94]. Vanadium pentoxide (V_2_O_5_) is a component of PM derived from fuel combustion as well as a source of occupation-related exposure in humans [95]. Sørensen et al. [95] indicate that vanadium and chromium (VI) detectable in PM_(2.5)_ have an effect on oxidative DNA damage in human lymphocytes, after reduction to chromium (III) in the cells. Since, outdoor PM and urinary 1-hydroxypyrene (PAH exposure marker) were synergistically associated with urinary MDA levels of schoolchildren, Bae et al. [96] concluded that exposure to PM air pollution and PAH can induce oxidative stress in schoolchildren. In addition, these authors found that urinary MDA levels are also associated with some metals bound to PM_10_ and PM_2.5_ suggesting that metals bound to PM are responsible, at least in part, for the oxidative stress [96]. The oxidized species arising from the reaction between ozone and lining fluid are involved in the signaling cascade of inflammatory cells into the lung and contribute to the acute bronchoconstrictor response and hyperresponsiveness observed in asthma on exposure to this pollutant [97, 98]. Furthermore, has been reported that ozone is able to induce apoptosis, DNA damage, and cytotoxicity on human alveolar epithelial type I-like cells and in mice exposed to ozone for 6 weeks [99, 100]. While, Ferecatu et al. [84] reported an antiapoptotic effect of PAH adsorbed on PM_2.5_ that in addition to the well-documented inflammatory response may explain the persistence of a prolonged inflammation state induced after pollution exposure and might delay repair processes of injured tissues in primary cultures of human bronchial epithelial cells. Chirino et al. [101] found ROS generation and decreased glutathione and the activity of the antioxidant enzymes, such as superoxide dismutase and glutathione reductase, in a human lung epithelial cell line exposed to PM_10_. Recently, it has been found that bus drivers exposed to PAH and volatile compounds displayed a higher level of DNA instability and oxidative damage than the controls and the incidence of oxidized lesions in lymphocyte DNA correlated with exposure to benzene. Moreover, those of the drivers with at least one variant of 8-oxoguanine glycosylate 1 (hOGG1) (Cys/Cys or Ser/Cys) allele tended to have higher oxidative DNA damage in lymphocyte than those with the wild genotype [102]. In addition, in the same year Delfino et al. [103] reported that PM (ranged from 0.25 to 2.5 *μ*m) and O_3_ were positively associated with exhaled nitrogen monoxide and that PM_0.25_, CO, and NO were positively associated with IL-6, while ROS were associated with both outcomes in elderly subjects enrolled.

## 7. Defense Mechanisms against ROS Formation

Antioxidants in the lung are the first line of defense against ROS [104]. The composition and quantity of antioxidants in respiratory tract lining fluids may represent an important determinant of individual responsiveness to air pollutants, but it should be thought of as a dynamic equilibrium with the antioxidant defenses within the epithelium and a more remote plasma pool [105]. Interestingly, the results obtained by Osburn and Kensler [106] demonstrated that the activation of transcriptional factor Nrf2 determines an upregulation of antioxidant enzymes that represents an adaptive response to face the exposure to oxidant pollutants providing a pivotal defense mechanism against environmental hazards, including various air pollutants. Successively, Rubio et al. [107] observed that Nrf2 does protect against benzene metabolites in human lung cells, and knockdown of Nrf2 greatly does enhance cytotoxicity and cell death associated with reduced glutathione levels and loss of inducibility of antioxidant response elements (ARE-driven) genes.

Although the interrelation among antioxidant levels in the respiratory tract, cellular and plasma levels are not well understood, it appears that the susceptibility of the lung to oxidative injury depends largely on its ability to upregulate protective scavenging systems. A recent review by Rubio et al. [108] indicates that air pollutants are Nrf2 pathway inductors which regulate the expression of cytoprotective and detoxifying enzymes as well as antioxidants having an important role in the defense against atmospheric pollutant-induced toxicity.

## 8. Conclusions

In conclusion, several experimental and epidemiological studies have proved exposure to air pollution to be an important determinant of overall pulmonary and cardiovascular risk damage and possibly have an influence on traditional risk factors. Although each environmental pollutant has its own mechanism of toxicity, most pollutants, like UFP, PM_2.5_, ozone, nitrogen oxides, and transition metals, are potent oxidants or capable of ROS production. Consequently, the promotion of oxidative stress has been identified as one of the most important mechanisms responsible for toxic air pollutant effects. Oxidative stress can trigger redox-sensitive pathways that lead to different biological processes like inflammation and cell death. Recently, Environmental Pollution Agency (EPA) revised the level of the 24-h PM_2.5_ standard to 35 *μ*g/m^3^, moved the 24-h PM_10_ standard from 75 at 150 *μ*g/m^3^, and revoked the annual standard, because available evidence generally did not suggest a link between long-term exposure to current ambient levels of coarse particles and health or welfare effects [109]. However, a vast number of data indicate that in general, smaller size fraction, containing higher concentration of PAH, transition metal, and semiquinones, has a higher ROS capacity and consequently should be capable to induce severe toxicological effects. Thus, change in the composition of this PM are likely to modify its health impact. Road traffic is known to vastly contribute to PM exposure. Traffic intensity and quality should then be important determinants of the qualitative characteristics of PM spread in the atmosphere. In addition, although the interrelation between antioxidant levels in respiratory and cardiovascular systems, cellular and plasma levels is not yet well understood; it appears that the susceptibility of target organs to oxidative injury largely depends on cell ability to upregulate protective scavenging systems such as Nrf2. This transcription factor does regulate the expression of numerous cytoprotective genes that detoxify reactive species playing an important role in the defense against atmospheric pollutant-induced toxicity.

However, many questions remain unanswered, but in the future, rapid developments in molecular biology, proteomics, and genomics will help to completely clarify the biological mechanisms involved in pulmonary and cardiovascular injuries caused by air pollution.
